# Occupational exposure to cadmium: protocol for a scoping review

**DOI:** 10.1136/bmjopen-2025-103361

**Published:** 2026-04-15

**Authors:** Marilia Silva Paulo, Carla Martins, Bruna Riesenberger, João Cordeiro, Renata Cervantes, Philippe Palmont, Rachna Bhoonah, Sophie Ndaw, Susana Viegas

**Affiliations:** 1 NOVA National School of Public Health, Public Health Research Centre, Comprehensive Health Research Centre, CHRC, REAL, CCAL, NOVA University Lisbon, Lisbon, Portugal; 2 Institute of Public Health, College of Medicine and health Sciences, United Arab Emirates University, Al Ain, Abu Dhabi, UAE; 3 H&TRC—Health & Technology Research Centre, ESTeSL—Escola Superior de Tecnologia e Saúde, Instituto Politécnico de Lisboa, 1990-096 Lisbon, Lisbon, Portugal; 4 National Agency for Food Environmental and Occupational Health and Safety, Maisons-Alfort, Île-de-France, France

**Keywords:** OCCUPATIONAL & INDUSTRIAL MEDICINE, PUBLIC HEALTH, Health & safety, Risk management

## Abstract

**Abstract:**

**Introduction:**

Cadmium is a metal that poses significant health risks, particularly in occupational environments where exposure can happen. The main objective of this scoping review is to review the cadmium exposure levels in the different occupational settings in the European Union (EU), considering the regulatory measures currently in place. The secondary objectives, depending on the availability of data, are (a) to identify the occupational settings where higher exposure levels occur, (b) to identify any geographical and temporal differences and trends within the EU and (c) to identify the most relevant co-exposures reported.

**Methods and analysis:**

A scoping review will be conducted in accordance with the Preferred Reporting Items for Systematic Reviews and Meta-Analyses Extension for Scoping Reviews reporting guidelines. Studies reporting quantitative occupational data on cadmium exposure obtained through human biomonitoring and/or air monitoring will be included. A descriptive analysis of the findings will be performed.

**Ethics and dissemination:**

This protocol for a scoping review does not require ethical approval as it is based on secondary data. The dissemination plan of the scoping review includes its publication in a scientific journal of reference, as it is expected that it will provide important knowledge to support ongoing and future occupational health interventions in the EU, at the technical and regulatory levels.

**Registration:**

This study is registered at the Open Science Framework (OSF), 7 April osf.f2w3h.

**Strengths and limitations of this study**

- This scoping review will identify and report the occupational exposure levels contributing to evidence-based policy making and regulation at the European level.
- The scoping review will summarise studies that provide quantitative data on human biomonitoring and/or air monitoring of occupational exposure to cadmium.
- The current protocol follows the Preferred Reporting Items for Systematic Review and Meta-Analysis Protocols guidelines, and the scoping review itself will follow the Preferred Reporting Items for Systematic Reviews and Meta-Analyses Extension for Scoping Reviews guidelines.

## Introduction

 Cadmium is a metal that poses significant health risks, particularly in occupational environments where exposure can happen.^1^,^3^ The target organs of this agent are the kidneys,^4^ bones and the respiratory system.^5^ Moreover, occupational exposure to metals such as cadmium (but also mercury, lead, arsenic, chromium and nickel) causes oxidative stress damage and can disrupt some proteins’ reproductive and activity.^6 7^ Although a wide range of adverse health effects are associated with chemical and environmental cadmium exposure, cancer remains the most relevant health concern driving regulatory actions within the European Union (EU).^8^ In Europe, specific regulations are intended to eliminate and reduce such exposure, aiming at protecting workers from the risks associated with exposure to carcinogens and supporting the implementation of measures to reduce exposure. These are the Directive 2022/431, also known as the Carcinogens, Mutagens and Reprotoxic Substances Directive,^9^ which provides an occupational exposure limit for cadmium and its inorganic compounds, with a combination of an airborne occupational exposure limit and a biological limit value. In addition, in the scope of the Registration, Evaluation, Authorisation and Restriction of Chemicals (REACH) regulation, there are 17 registered substances for cadmium and its compounds.^10 11^ Cadmium and its compounds are not listed in Annex XIV of REACH (“Authorisation List”), meaning that there are no authorised uses for cadmium and its compounds. Some substances (cadmium, cadmium oxide, cadmium sulphide, cadmium sulphate, cadmium chloride, cadmium fluoride, cadmium carbonate, cadmium hydroxide and cadmium nitrate) have been identified as substances of very high concern and are included in the REACH Candidate list for authorisation.^10^ In addition, there are restricted uses under Annex XVII of REACH, such as mixtures and articles produced from plastic material, paints, plating metallic articles or components of these articles used in certain sectors/applications, metal beads and other metal components for jewellery making.^12^ These restrictions mainly aim to protect the general population, but indirectly also prevent workers’ exposure.

However, even with all these regulations in place in the EU, occupational exposure still occurs, particularly in occupational settings where substitution is still not possible. Consequently, implementing new and improved risk management would be of extreme relevance to mitigate exposure.^213^,^15^

The occupational settings where cadmium exposure is still expected to occur include processes involving heating cadmium-containing materials, such as smelting and electroplating. Occupations reported with the highest exposure levels include smelting zinc and lead ores, welding or remelting cadmium-coated steel, working with solders that contain cadmium, and producing, processing and handling cadmium powders. Recycling operations, such as the ones related to nickel-cadmium batteries, may also generate significant additional occupational exposure, as the circular economy EU goals tend to increase the number of workers involved in recycling operations.^10 11^ Moreover, most of these occupational settings are characterised by the presence of many other metals and other groups of substances. For example, during electroplating, workers can be exposed to nickel, chromium hexavalent and Per- and polyfluoroalkyl substances;^16^ in battery production and recycling of e-waste, lead is a substance of concern, as is cadmium.^17 18^ In the case of welding processes, substances present in welding fumes are determined by both the composition of the ligands and that of the surfaces being welded.^19 20^

In these occupational settings, exposures occur essentially by inhalation but also by ingestion through hand-to-mouth contact due to the contamination of surfaces, equipment and even personal protection equipment.^21 22^

Considering the routes of occupational exposure, a combination of techniques (eg, air monitoring and biological monitoring) is typically used to assess exposure to cadmium. Personal and stationary sampling devices are commonly used in workplaces to measure cadmium levels in air.^2 18^ Biological monitoring is also widely used to evaluate long-term cadmium exposure, as the body retains cadmium for extended periods.^23 24^ The existence of a biological limit value also supports the use of biomonitoring as an exposure assessment tool.^21^ Additional exposure assessment methods, such as surface and dermal wipe sampling, help determine the presence of cadmium on work surfaces, tools, protective equipment and skin. Job exposure matrices, surveys and questionnaires about workers’ job histories, operational conditions and risk management measures in place can also contribute to exposure assessment by providing supplementary data on workplace conditions and exposure scenarios.^2 18^ All these exposure assessment methods should be considered as complementary approaches since all provide different information on exposure levels, exposure routes and determinants of exposure, and provide insights on the risk management measures to implement or improve.

**Figure 1:**
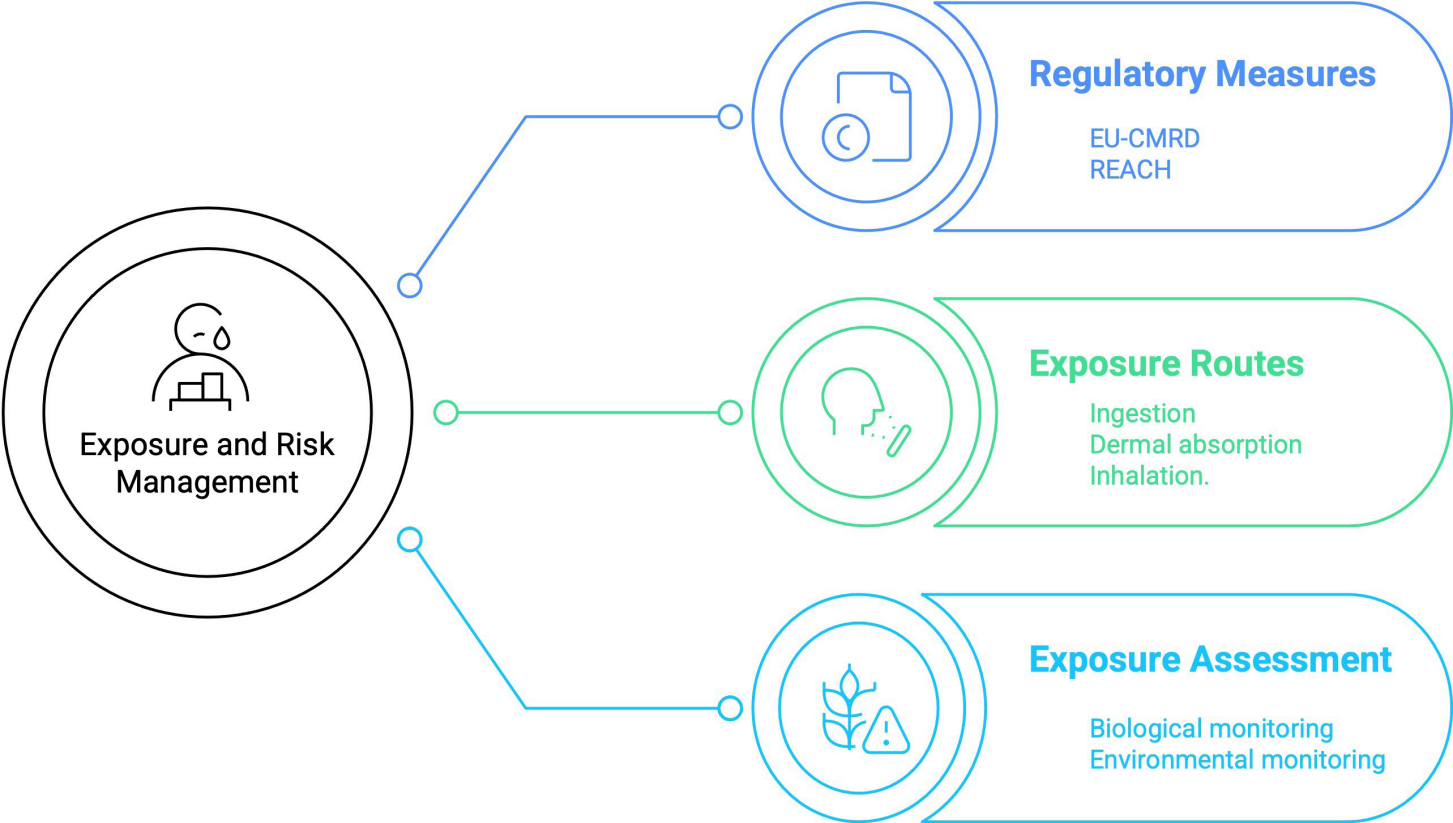
Conceptual map of occupational exposure to cadmium. EU-CMRD, European Union-Carcinogens, Mutagens and Reprotoxic Substances Directive; REACH, Registration, Evaluation, Authorisation and Restriction of Chemicals.

Based on the above-identified knowledge gaps and the need to have a comprehensive understanding of cadmium occupational exposure within the EU, conducting a scoping review was considered both relevant and necessary to develop (figure 1).

Therefore, the main objective of the scoping review proposed in this protocol is to review the cadmium exposure levels in the different occupational settings in the EU, considering the regulatory measures in place. The secondary objectives, depending on the availability of data, are (a) to identify the occupational settings where higher exposure levels occur, (b) to identify any geographical and temporal differences and trends within the EU and (c) to identify the most relevant co-exposures reported.

## Methods

### Protocol and registration

The current protocol was written following the Preferred Reporting Items for Systematic Review and Meta-Analysis Protocols^25^ and Preferred Reporting Items for Systematic Reviews and Meta-Analyses Extension for Scoping Reviews reporting guidelines^26^ (online supplemental material). The scoping review manuscript will be organised around the core scoping review stages (identifying the research question; identifying relevant evidence; selecting evidence; charting the data and synthesising and reporting results), consistent with widely accepted scoping review practice. The protocol was registered at the Open Science Framework (OSF) at osf.io/f9krw,^1^ after the completion of the generalised registration form for systematic reviews.^27^ The initiation date of the review was April 2025, and its end is planned for January 2026.

### Eligibility criteria

The eligibility criteria for this scoping review align with its defined objectives. Studies will be considered eligible if they reported quantitative data on human biomonitoring (HBM) and/or air monitoring of occupational exposure to cadmium, were conducted in EU countries, were written in English and were published on or after the 1st of January 2010. Table 1 outlines the inclusion and exclusion criteria.

**Table 1:** Inclusion and exclusion criteria

Inclusion criteria	Exclusion criteria
Studies assessing occupational exposure.	Studies describing exposure in the general population.
Analytical studies including original data (they might include interventional studies, ecological studies, cross-sectional studies, prospective and retrospective studies).	Reviews, editorials, letters-to-editor, commentaries and conference abstracts with incomplete data on exposure or methods.
Studies reporting occupational exposure data from EU countries.	Studies reporting occupational exposure data from non-EU countries.
Studies including quantitative data on cadmium exposure through air monitoring, biomonitoring or other exposure assessment approaches.	Studies lacking quantitative data on cadmium exposure.
Description of the occupational setting.	Absence of information on the occupational setting.
Written in English.	Not written in English.
Studies published between 1 January 2010, and the date of the search strategy implementation.	Studies published before 1 January 2010.

EU, European Union.

### Information sources

The information sources for this scoping review will include the electronic databases PubMed (MEDLINE), Scopus and Web of Science. This scoping review will not include grey literature.

The final search results will be uploaded into Covidence, a software designed to better manage systematic reviews. Duplicate records will be automatically identified and removed when uploading the search results in the .ris files format.

### Search

The search strategy will be specifically designed for this scoping review, aiming to comprehensively capture all relevant studies addressing the defined objectives. Initial search terms will be developed to identify studies conducted in occupational settings that report HBM and/or air monitoring data on cadmium compounds. Additional exposure assessment approaches will be considered if quantifiable data is provided. Broad search terms will be included, such as “cadmium”, “workplace”, “worker” and “occupation”. Medical Subject Headings terms will be used where applicable to improve search precision. Given the multiple secondary objectives aimed at advancing understanding of occupational exposure to cadmium, the search strategy has been designed to be comprehensive rather than overly specific to ensure no potentially eligible studies are missed. The proposed search strategy is in the online supplemental material.

The search will be updated prior to finalising the synthesis of evidence to ensure that newly published eligible papers will be identified and included.

### Selection of sources of evidence

The scoping review will be performed by a group of nine researchers. Covidence will be used for screening eligible studies. The reviewers will screen titles and abstracts independently. All titles and abstracts will be screened by at least two independent reviewers. The inclusion and exclusion criteria will be specified within COVIDENCE. Before initiating the screening process, the research team will convene to review the COVIDENCE setup and harmonise the screening process, ensuring consistency in study selection. Conflicts will be solved by a third reviewer who is not involved in the initial screening of that reference. A meeting to discuss conflict resolution will be held in advance to ensure harmonisation among reviewers. During the first stage of screening, only the authors, title and abstract authorship information will be visible to reviewers. On the second stage, full text screening, the same procedure and criteria will be applied.

References of all included studies will be manually reviewed to identify any further relevant publications.

### Data items

A data extraction sheet (data-charting form) will be developed and piloted until there is convergence and agreement by all the team members.

Instructions regarding what is expected for each variable will be developed during the piloting stage. Reviewers will extract all the data that might be important to answer the objectives. A designated section will be included in the data extraction form to complement data extraction with relevant excerpts or supporting sentences from the studies. Data will be extracted on the type of study, country, EU region, year of exposure assessment campaign, occupation, sector of activity, type of exposure assessment approach used and samples considered, exposure levels reported (mean, range of values), number of participants, sex, age, use of Personal Protection Equipment (PPE), risk management measures described and co-exposures reported and/or mentioned in the publication (even if not having exposure data).

A meeting to standardise and harmonise the details of data extraction will be held prior to its start. Data extraction will be completed by one author and reviewed by another. In the case of disagreement, a meeting will be held, and priority will be given to the most comprehensive extraction form.

When data is not reported or the category predefined in the data extraction sheet is not applicable, this will be described in the synthesis of results and appropriate tables. In the case of multiple articles from the same underlying study, all studies will be documented, and data from the most completely described one will be reported.

### Critical appraisal of individual sources of evidence

Knowledge synthesis methods will be used to critically appraise the included studies. While analytical studies are anticipated to be included, considerable variability in study design is also expected. A combination of appraisal domains may be applied, drawing on existing validated tools. The tool proposed by Pega *et al*^28^ for assessing the risk of bias in studies estimating the prevalence of exposure to occupational risk factors is expected to be the most appropriate for this review.^28^

### Synthesis of results

A synthesis of results will be presented, describing exposure levels across different occupational settings where cadmium exposure occurs in Europe. The review will also describe these settings and associated levels of exposure to identify possible data gaps. A detailed qualitative synthesis will be conducted based on reported exposure levels and identified exposure determinants.

If the data allows, qualitative synthesis and/or sensitivity analysis will be conducted according to the United Nations geoscheme for European subregions, considering the cadmium occupational exposures reported for Eastern Europe, Northern Europe, Southern Europe and Western Europe.^29^

The comparison between the occupational exposure limits of cadmium and the exposure levels reported in the included studies will also be described.

## Ethics and dissemination

Ethical approval is not required for this scoping review.

The dissemination plan of the proposed study is to publish the main findings in a scientific journal of reference. The main data files will be published as online supplemental materials.

## Supplementary material

10.1136/bmjopen-2025-103361 online supplemental file 1

10.1136/bmjopen-2025-103361 online supplemental file 2

## Data Availability

No data are available.
