# Supplementary material for: Occupational exposure to cadmium: protocol for a scoping review
Source: BMJ Open. 2026 Apr 15;16(4):e103361. doi: 10.1136/bmjopen-2025-103361 (PMC13084944; doi:10.1136/bmjopen-2025-103361)
Supplement: online supplemental file 1 [file bmjopen-16-4-s001.docx]

**SUPPLEMENTARY MATERIAL**

| **Database** | **Search String** | **Filters applied** | **Number of records** | **Date** |
| --- | --- | --- | --- | --- |
| PubMed | (((((“Workplace”[Mesh]) OR (workplace[Title/Abstract])) OR (worker[Title/Abstract])) OR (“occupational exposure”[Mesh])) OR (“occupational exposure$”[Title/Abstract])) AND ((“Cadmium”[Mesh]) OR (cadmium[Title/Abstract])) Filters: English, from 2010/1/1 – 3000/12/12 | From 2010; English language | 486 | 09/12/2024 |
| Scopus | ( ( TITLE-ABS-KEY ( workplace ) ) OR ( TITLE-ABS-KEY ( worker ) ) OR ( TITLE-ABS-KEY ( “occupational exposure” ) ) ) AND ( ( TITLE-ABS-KEY ( cadmium ) ) ) AND PUBYEAR > 2009 AND PUBYEAR < 2026 AND ( LIMIT-TO ( LANGUAGE , “English” ) ) | From 2010; English language | 1528 | 09/12/2024 |
| Web of Science | #10 AND #7  #9 OR #8  TS=(“cadmium”)  TI=(“cadmium”)  #1 OR #4 OR #3 OR #5 OR #6  TI=(“occupational exposure”)  TS=(“occupational exposure”)  TI=Workplace (Title)  TI=Worker (Title)  TS=Worker (Topic)  TS=Workplace (Topic) | From 2010; English language | 665 | 09/12/2024 |
