# Supplementary material for: Occupational exposure to cadmium: protocol for a scoping review
Source: BMJ Open. 2026 Apr 15;16(4):e103361. doi: 10.1136/bmjopen-2025-103361 (PMC13084944; doi:10.1136/bmjopen-2025-103361)
Supplement: Supplementary file 3 [file bmjopen-2025-103361.reviewer_comments.pdf]

PEER REVIEW HISTORY

BMJ Open publishes all reviews undertaken for accepted manuscripts. Reviewers are asked to complete a checklist review form and are provided with free text boxes to elaborate on their assessment. These free text comments are reproduced below.

ARTICLE DETAILS

Title (Provisional)

Occupational exposure to Cadmium: Protocol for a Scoping Review

Authors

Paulo, Marilia Silva; Martins, Carla; Riesenberger, Bruna; Cordeiro, João; Cervantes, Renata; Palmont, Philippe; Bhoonah, Rachna; Ndaw, Sophie; Viegas, Susana

---

VERSION 1 - REVIEW

**Reviewer**	**1**
**Name**	**Fahim, Yosri A.**
**Affiliation**	**Galala University**
**Date**	**19-Jul-2025**
**COI**	**None**

---

1. Abbreviations:All abbreviations (e.g., EU-CMRD, REACH, SVHC) should be spelled out at first use in both the abstract and main text for clarity, especially for an international readership

2. you should read and cited theses relevant references, they will enhance the introduction section:

-DOI: 10.60829/jchr.2024.19934

-doi.org/10.5696/2156-9614-10.27.200903

3. In table 2 line 21 1n2 22, please recheck this sentence : Filters: English, from 2010/1/1 – 3000/12/12

4. The conclusion section is missing, please add arelavent conclusion

5. it is prefereable if you include relavent and schematic figure to enhance it

---

Reviewer **2**

Name **Tawiah, Philip**

Affiliation **University of Health and Allied Sciences, Department of Pharmacognosy and Herbal Medicine, School of Pharmacy**

Date 08-Aug-2025

COI None

Manuscript: Occupational exposure to Cadmium: Protocol for a Scoping Review

General comments

1. Authors should recraft the scoping review using recommended framework and format.

2. Without the framework and format, it is difficult to put the work into proper perspective. When the abstract contains a section like participants, then it makes it even more confusing.

3. There is no proper justification in the introduction.

4. The language and flow of English should also be looked at. For instance, the scoping review is to review …

5. Setting in the abstract can never refer to occupational settings. Authors need to relook at the whole manuscript again

---

Reviewer 3

Name Martinez Jarreta, Begoña

Affiliation University of Zaragoza, Occupational and Forensic Medicine

Date 29-Nov-2025

COI None

The article presents the protocol on which a scoping review of occupational exposure to cadmium will be based. It therefore addresses a topic of considerable importance, as the authors aim to review cadmium exposure levels across different occupational settings within the European Union, taking into account the regulatory measures that have been implemented. Various stated secondary objectives are also considered.

Several improvements are needed for the protocol acceptance for publication. These are:

1) Given that the protocol corresponds to a scoping review, it currently does not include a search of grey literature—an aspect that would be highly advisable in this case, particularly since the review seeks to examine the implementation of regulatory measures across EU different regions and countries. In this regard, MECIR and Cochrane standards recommend that authors: “Search relevant sources of grey literature, such as reports, dissertations, theses, databases, and conference abstract repositories. Searches for studies should be as comprehensive as possible to reduce the risk of publication bias and to identify as much relevant evidence as possible.”

2) Regarding the inclusion and exclusion criteria, the types of articles to be included have not been described in sufficient detail. The protocol mentions only “analytical studies,” but the specific study designs to be included within this category should be explicitly stated.

3) The types of articles to be included have not been described in sufficient detail in the inclusion and exclusion criteria. The protocol mentions only 'analytical studies', but the specific study designs to be included within this category should be explicitly stated.

4) The exclusion criteria use the term 'conference proceedings with insufficient information'; however, this wording is vague and should be defined precisely. It would be more appropriate to specify exclusion due to missing or incomplete data/essential information.

5) The protocol should also include the rules that will be followed when information is missing or incomplete in a study (e.g. coded as 'not reported' or, if ambiguous, 'unclear').

6) Similarly, the data extraction section should explain how data will be handled in cases where multiple articles have been published from the same underlying study (commonly referred to as related or companion studies).

7) Regarding ethical requirements, the protocol should explicitly state whether ethical approval is required for conducting a scoping review at the authors’ institution.

8) In the conclusión:Restate the primary and secondary research questions that guide the inquiry, providing a concise summary of their scope and relevance, Mention: Possible gaps found in the existing literature that the scoping review aims toaddress, emphasising the limitations and unmet needs identified in the preliminary analysis. Articulate how the present scoping review is positioned to advance the field, specifying the contributions it aims to offer and future research directions.

VERSION 1 - AUTHOR RESPONSE

Dear Dr. Yosri A. Fahim, Galala University,

We appreciate the time taken to revise our manuscript. Thank you.

The points below provide information on how we have addressed the comments made.

1. Abbreviations: All abbreviations (e.g., EU-CMRD, REACH, SVHC) should be spelled out at first use in both the abstract and main text for clarity, especially for an international readership

Author‘s response: We have revised the manuscript accordingly.

2. you should read and cited theses relevant references, they will enhance the introduction section:

-DOI: 10.60829/jchr.2024.19934

-doi.org/10.5696/2156-9614-10.27.200903

Author's response: we have included these references in the manuscript and its importance related to oxidative stress.

3. In table 2 line 21 1n2 22, please recheck this sentence : Filters: English, from 2010/1/1 – 3000/12/12

Author's response: Table 2 was moved to Supplementary material as an indication from the Editor. We did not correct the sentence as it is exactly the way it should be placed in PubMed advanced search.

4.The conclusion section is missing, please add relevant conclusion

Author's response: Dear Reviewer, we followed the PRISMA-P guidelines and checklist and in a review protocol there are no conclusion section. We have added the PRISMA-P checklist completed with the pages where information can be found. Please see Supplementary material 1.

5. it is preferable if you include relevant and schematic figure to enhance it

Author's response: Thank you for the suggestion. Please see Figure 1.

Dear Dr. Philip Tawiah, University of Health and Allied Sciences

We appreciate the time taken to revise our manuscript. Thank you.

The points below provide information on how we have addressed the comments made, one by one.

1. Authors should recraft the scoping review using recommended framework and format.

Author's response: Please see page 4, under *protocol and registration*. This protocol is following PRISMA-P guidelines and the scoping review itself will follow PRISMA-ScR. Moreover, we submitted the PRISMA-P checklist completed as a supplementary material.

2. Without the framework and format, it is difficult to put the work into proper perspective. When the abstract contains a section like participants, then it makes it even more confusing.

Author's response: We have amended the abstract in accordance. Please see *abstract* section.

3. There is no proper justification in the introduction.

Author's response: Please see page 4, where you can read "Based on the above identified knowledge gaps, and the need to have a comprehensive understanding of cadmium occupational exposure within the EU, conducting a scoping review was considered both relevant and necessary to develop."

4. The language and flow of English should also be looked at. For instance, the scoping review is to review …

Author's response: We have revised the manuscript accordingly.

5. Setting in the abstract can never refer to occupational settings. Authors need to relook at the whole manuscript again

Author's response: We have amended the abstract in accordance. Please see *abstract* section.

Dear Prof. Begoña Martinez Jarreta, University of Zaragoza

We appreciate the time taken to revise our manuscript. Thank you.

The points below provide information on how we have addressed the comments made, one by one.

The article presents the protocol on which a scoping review of occupational exposure to cadmium will be based. It therefore addresses a topic of considerable importance, as the authors aim to review cadmium exposure levels across different occupational settings within the European Union, taking into account the regulatory measures that have been implemented. Various stated secondary objectives are also considered.

Several improvements are needed for the protocol acceptance for publication. These are:

Given that the protocol corresponds to a scoping review, it currently does not include a search of grey literature—an aspect that would be highly advisable in this case, particularly since the review seeks to examine the implementation of regulatory measures across EU different regions and countries. In this regard, MECIR and Cochrane standards recommend that authors: "Search relevant sources of grey literature, such as reports, dissertations, theses, databases, and conference abstract repositories. Searches for studies should be as comprehensive as possible to reduce the risk of publication bias and to identify as much relevant evidence as possible."

Author's response: Dear reviewer, thank you for pointing out this. Please let me explain that we considered to include grey literature but our search string retrieves a high number of records and we decided that we will not advance. We added the information about the non-inclusion of grey literature. Please see page 5, under *information sources*.

2) Regarding the inclusion and exclusion criteria, the types of articles to be included have not been described in sufficient detail. The protocol mentions only "analytical studies," but the specific study designs to be included within this category should be explicitly stated.

Author's response: We added this information. Please see page 5, Table 1.

3) The types of articles to be included have not been described in sufficient detail in the inclusion and exclusion criteria. The protocol mentions only 'analytical studies', but the specific study designs to be included within this category should be explicitly stated.

Author's response: We added this information. Please see page 5, Table 1.

4) The exclusion criteria use the term 'conference proceedings with insufficient information'; however, this wording is vague and should be defined precisely. It would be more appropriate to specify exclusion due to missing or incomplete data/essential information.

Author's response: We corrected this information to "conference abstracts with incomplete data on exposure or methods". Please see page 5, Table 1.

5) The protocol should also include the rules that will be followed when information is missing or incomplete in a study (e.g. coded as 'not reported' or, if ambiguous, 'unclear').

Author's response: Thank you for the suggestion, we added. Please see page 6, under *data items*.

6) Similarly, the data extraction section should explain how data will be handled in cases where multiple articles have been published from the same underlying study (commonly referred to as related or companion studies).

Author's response: Thank you for the suggestion, we added. Please see page 6, under *data items*.

7) Regarding ethical requirements, the protocol should explicitly state whether ethical approval is required for conducting a scoping review at the authors' institution.

Author's response: Suggestion implemented. Please see page 8, under *ethics and dissemination*.

8) In the conclusión: Restate the primary and secondary research questions that guide the inquiry, providing a concise summary of their scope and relevance, Mention: Possible gaps found in the existing literature that the scoping review aims to address, emphasising the limitations and unmet needs identified in the preliminary analysis. Articulate how the present scoping review is positioned to advance the field, specifying the contributions it aims to offer and future research directions.

Author's response: Dear Reviewer, we followed the PRISMA-P guidelines and checklist and in a review protocol there are no conclusion section. We have added the PRISMA-P checklist completed with the pages where information can be found. Please see Supplementary material 1.
